# Treatment of a coronal shear injury of the trochlea using a modified hedgehog-based technique through an anterior neurovascular interval approach: A case report

**DOI:** 10.1016/j.ijscr.2021.106211

**Published:** 2021-07-16

**Authors:** M. Koëter, P.P.W. van Hugten, P.J. Emans, J.A. Ten Bosch

**Affiliations:** a Departement of Trauma Surgery, Maastricht University Medical Center, Maastricht, the Netherlands; b Department of Orthopaedic Surgery, Maastricht University Medical Center, Maastricht, the Netherlands

**Keywords:** Humeral trochlear fracture, Anterior neurovascular approach, Modified hedgehog-based technique, Case report

## Abstract

**Introduction and importance:**

Surgical treatment for dislocated trochlear shear injuries is recommended due to its articular nature. However, the surgical exposure is often limited and large cartilaginous fragments with lack of subchondral bone stock makes a stable fixation technically challenging. Rapid swelling of the traumatized cartilage induces a size misfit between the cartilage of the fragment and the defect site. The use of a “modified hedgehog”-based technique might be a solution in these cases. However, this technique has only been described in chondral shear lesions of the knee.

**Case presentation:**

A fifteen-year-old boy fell out of the still rings during gymnastics at school and suffered trauma to his elbow. A CT-scan of the right elbow showed a coronal shear injury of the trochlea with accompanied lateral condyle fracture of the humerus. The patient was treated throughout an anterior neurovascular approach with a modified hedgehog-based technique with triple fixation by creating an interlocking match of the cartilage, application of fibrin glue and additional screw fixation.

**Clinical discussion:**

The anterior neurovascular interval approach provides a clear exposure of the fracture site which is useful for anatomic reduction and triple fixation. The subsequent modified hedgehog-based technique has only been described once in children with shear-off chondral fragments of the knee, without additional screw fixation. Adding a screw fixation of the fragment creates some collateral damage to the cartilage and might not be necessary in future cases.

**Conclusion:**

The anterior neurovascular approach seems elegant and provides adequate exposure. Furthermore, a modified hedgehog-based technique delivers a stable triple fixation of the osteochondral fragment.

## Highlights

• Surgical treatment of coronal shear injury of the trochlea is challenging.
• Anatomic reduction and stable fixation of a coronal shear injury is essential.
• The anterior neurovascular approach provides extensive exposure of the fracture site.
• A modified hedgehog-based technique delivers stable triple fixation.

## 1 Introduction

In 1853 Laugier was the first to describe an isolated fracture of the trochlea, afterwards known as a Laugier fracture [1]. Humeral trochlear fractures occur rarely since the humeral trochlea has no muscular or ligamentous attachments and because of its location within the olecranon fossa, making it less susceptible to direct trauma. Therefore, trochlear fractures are seldomly isolated but usually accompanied with other injuries like ligamentous injuries or fractures of the capitellum, radial head or olecranon [1], [2], [3]. The mechanism of injury in case of an isolated injury to the trochlea is still under debate. The fracture might be a result of axial loading with the elbow in extension, like a fall on an outstretched arm. A role of varus stress combined with axial loading has also been suggested [2].

**Fig. 1:**
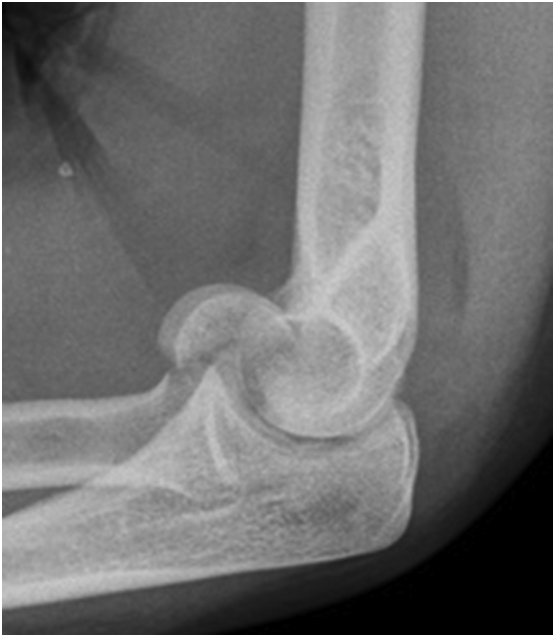
Double arc sign on lateral view.

Initial evaluation after injury should include plain radiographs. On an anteroposterior view only, the fracture can easily be missed. However, on a lateral view a half-moon shaped fragment or a double arc sign is typically present (Fig. 1). Computed tomography (CT) is recommended for further diagnostics and preoperative planning [4], [5].

(Case courtesy of Dr. Benoudina Samir, Radiopaedia.org, rID: 67687)

Surgical treatment for dislocated trochlear fractures is recommended due to its articular nature. Failure in anatomic reduction and fixation leads to arthrosis, functional impairment and potential instability [5]. However, the optimal surgical exposure and fixation technique are debatable. The surgical exposure is often limited and large cartilaginous fragments with lack of subchondral bone stock makes a stable fixation technically challenging [4], [5].

In literature, the most commonly used surgical approach for displaced trochlear fracture is a medial approach. A lateral approach and even a posterior approach with olecranon osteotomy are also described [3], [4], [5]. However, these approaches all require excessive soft tissue dissection and often offer limited exposure of the injured site. A recent article of Yang et al. describes an anterior neurovascular interval approach providing some advantages such as a simple anatomical layer, moderate dissection of surrounding tissue with fewer side injuries and a clear operative field [6].

After anatomical reduction, the next step is fixation of the fracture. Since trochlear fractures are often in the coronal plane, anteroposterior fixation is preferable. In many described cases in literature, this is achieved by using compression screws, Kirschner wires or headless Hebert screws [2], [3], [4], [5]. However, this fixation causes collateral damage to the cartilage and, in case of a small subchondral bone fragments, stable fixation is a challenge.

Adding to this challenging fixation, rapid swelling of the traumatized cartilage induces a size misfit between the cartilage of the fragment and the defect site [7]. This swelling is induced by the two major components of the extracellular matrix of articular cartilage, specifically collagen and proteoglycan [8]. These two components induce a tension balance between the collagen fibers resisting tensile strain by the osmotic swelling pressure due to the proteoglycan induced water uptake [9]. When the integrity of the collagen fibers is disrupted due to trauma the cartilage starts to swell by the increased water uptake [10].

Overcoming this swelling misfit and providing additional stability, the use of a “modified hedgehog” -based technique [7] might be a solution in these cases. In this technique the cartilage swelling of the fragment and the defect edges are trimmed in an angle to create an interlocking match. Note that stability due to this interlocking match relies on the exact fit of the fragment and the defect.

In this report we present, by following the SCARE 2020 guidelines [11], the first case using a modified hedgehog-based technique in a coronal shear injury of the trochlea with accompanied lateral condyle fracture of the humerus in an adolescent patient.

## 2 Case description

### 2.1 Patient information and clinical findings

A fifteen-year-old boy fell out of the still rings during gymnastics at school. He suffered trauma to both elbows with pain and loss of function. He had no relevant past medical or surgical history.

### 2.2 Diagnostic assessment

**Fig. 2:**
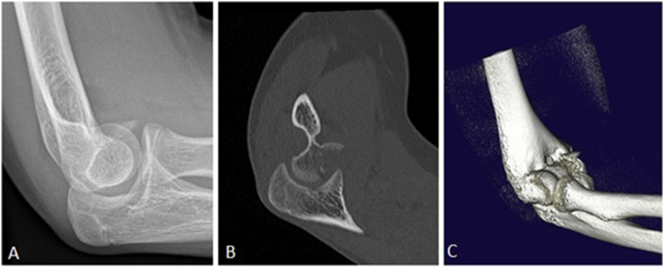
A: Lateral view of right elbow with suspicion of a coronal shear injury. B: Sagittal CT scan of coronal shear injury. C: 3D reconstruction of the joint.

Radiographs showed a non-displaced supracondylar fracture on the left side, and a lateral condyle fracture with also suspicion of a dislocated coronal shear injury on the lateral view of his right elbow (Fig. 2a). The left supracondylar fracture was treated conservatively with an above-elbow cast. A CT-scan of the right elbow showed a coronal shear injury of the trochlea with accompanied lateral condyle fracture of the humerus (Fig. 2b and c).

### 2.3 Therapeutic intervention

Open reduction and internal fixation of the lateral condyle fracture as well as the trochlear injury were planned. The surgical intervention was performed by the third and fourth author.

**Fig. 3:**
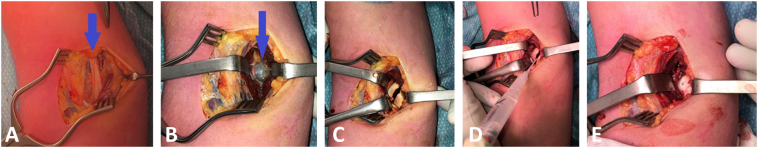
A: S-shaped incision, identifying median nerve (arrow) and brachial artery. B: Bulging joint capsule with haemarthros (arrow). C: Overview of the coronal shear fracture. D: Filling defect site with fibrin glue (Tissucol, Baxter, The Netherlands). E: Reduction of fracture and speedtip (1.5 mm) screw fixation.

The patient was positioned supine on the operating table with his right arm on a radiolucent table. Patient received cefazoline prophylaxis preoperatively. First, closed reduction and percutaneous cannulated lag screw fixation of the lateral condylar fracture with use of image intensifier was performed. Second, an anterior neurovascular interval approach was initiated [6]. A volar S-shaped incision was made from medial (proximal) to lateral (distal), with an incision throughout the bicipital aponeurosis. The brachial artery and median nerve (Fig. 3A) were identified.

Entering through this neurovascular interval, the brachial muscle was approached. By splitting the brachial muscle, the joint was reached. In Fig. 3B, the bulging joint capsule is apparent due to extensive haemarthros.

A longitudinal arthrotomy with release of hematoma was performed. After rinsing the joint, the coronal shear fracture was visualized (Fig. 3C). The dislocated osteochondral injury was measured as 15 × 15 mm.

The osteochondral fragment was removed. Swelling of the chondral tissue of the shear-off fragment after trauma calls for trimming of the edges in an angle of approximately 60–80° of both the edges of the fragment as well as the defect in order to create an interlocking match [7]. After debriding the subchondral bone and abrading the defect site, removing any fibrous tissue, the defect site was filled with fibrin glue (Tissucol, Baxter, The Netherlands) and the sheared off fragment was placed back, resulting in a congruent articulating surface (Fig. 3D) and a stable interlocking match.

One 14 mm speedtip screw (1.5 mm, Medartis) was placed subchondral in order to deliver additional compression and stability (Fig. 3E).

**Fig. 4:**
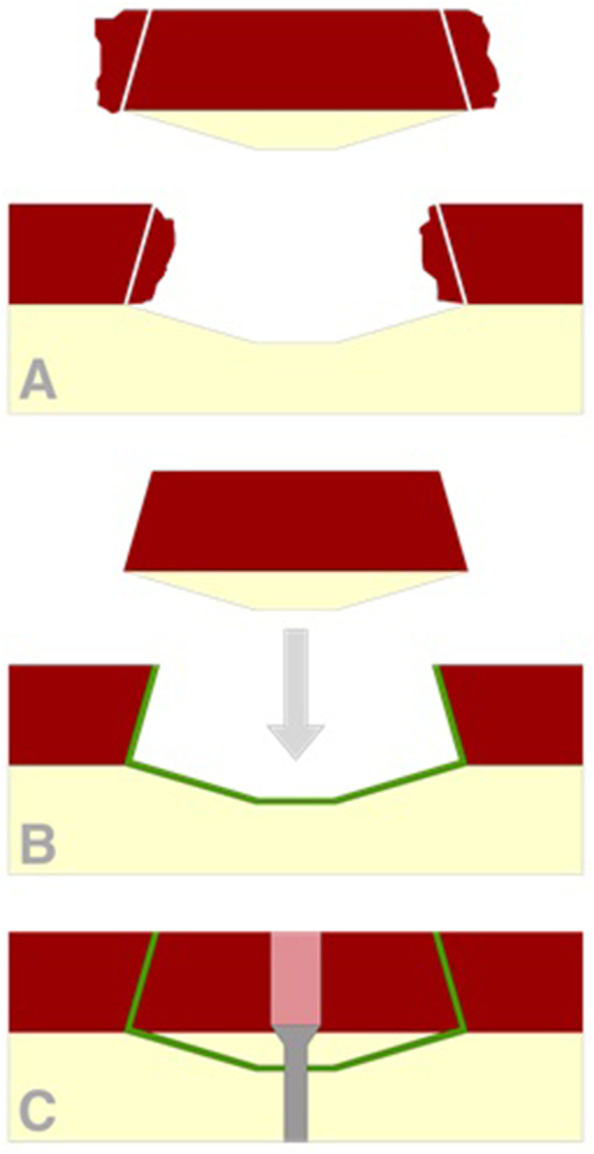
Illustration of the triple fixation of the sheer of osteochondral fragment by the use of interlocking angles of the cartilage tissue, fibrin glue and screw fixation. A: Cartilage swelling allows trimming of both the fragment and defect edges in an angle to create an interlocking match. B: After application of fibrin glue (green) in the defect site, the fragment is placed back. C: After steps A and B, the osteochondral fragment is fixed using screw fixation. (For interpretation of the references to colour in this figure legend, the reader is referred to the web version of this article.)

By combining these three fixation techniques, interlocking cartilage, fibrin glue and screw fixation, a stable fixation of the challenging osteochondral fragment was achieved (Fig. 4).

**Fig. 5:**
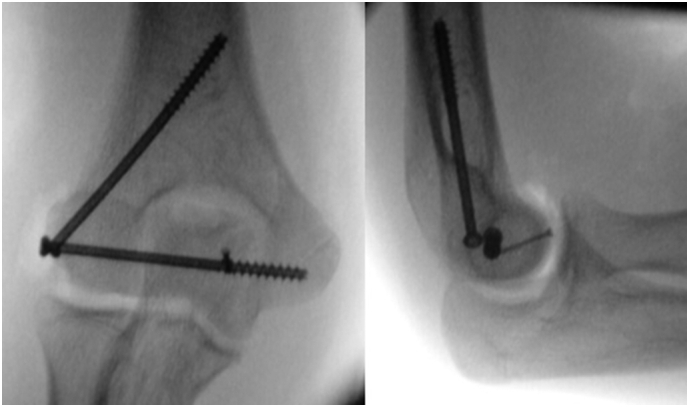
Perioperative view on image intensifier.

After checking the reduction and fixation with the image intensifier (Fig. 5), the joint capsule was closed, followed by the subcutaneous tissue and skin. A full range of motion was reached after surgery. We supplied a protective above-elbow cast for two weeks.

### 2.4 Follow-up and outcomes

Two weeks after surgery, the cast was removed and radiographs were acquired for follow up. At this point, the patient was encouraged to exercise without heavy lifting.

After three months follow up, patient reached a full range of motion of the right elbow, with 140 degrees of flexion, full extension, 85 degrees of pronation, and 80 degrees of supination. There were no adverse events and the patient was very satisfied with the functional results.

## 3 Discussion

The present paper describes a case report of a fifteen-year-old boy with a coronal shear injury of the trochlea with accompanied lateral condyle fracture of the humerus which was treated with percutaneous lag screw fixation and a modified hedgehog-based technique by an anterior neurovascular interval approach.

The anterior neurovascular interval approach provided clear exposure of the coronal shear injury of the trochlea, which is especially important when performing the modified hedgehog-based technique with triple fixation of osteochondral fragment. A clear overview of the osteochondral fragment is necessary to measure and create the interlocking angles as well as for compression in an anterior to posterior direction. Furthermore, with this anterior approach, all notable anatomic structures are easily exposed and therefore less prone to accidental injury [6]. In case of concomitant capitellum fracture, a second anterolateral interval as described by Tanwar et al. [12] can be obtained through the same S-shaped skin incision in order to expose the anterior portion of the capitellum.

Stable fixation of small osteochondral fragments is challenging. In this case report, we described a modified hedgehog-based technique with triple fixation by the use of interlocking angles of the cartilage tissue, fibrin glue and screw fixation. The modified hedgehog technique provided by Jeuken et al. [7] in 2019 showed promising results in children with shear-off chondral fragments of the knee with no loosening of the fragment, no pain and complete return to sports with full range of motion. Based on these results, the osteochondral fragment was fixated with interlocking angels and fibrin glue. These interlocking angles prevents rotation of the fragment, however a 1.5 mm subchondral screw fixation was performed for additional compression and stability since the bony aspect of the osteochondral fragment was quite bulky. However, transchondral screw placement damages the still intact cartilage of the fragment. Furthermore, there is available literature suggesting a fibrin sealant alone versus internal fixation is sufficient in osteochondral defects described in an animal study [13] and a study of osteochondral fractures of the digits [14]. Therefore, in future cases it might not be necessary to perform additional screw fixation.

Although the evidence for the use of fibrin glue to treat large chondral fractures is limited, its application in the treatment of osteochondral defects is widely accepted [15]. The drawback of these allogenic fibrin-based adhesives is the derivation of them from human blood, thus carrying the risk of contamination and inducing an immune response due to foreign body exposure [16]. To minimize these risks, the use of autologous fibrin-based sealants is advocated [17]. Alternatively, non-biologic glues like cyanoacrylate and aldehyde-based adhesives are available. However, these have shown to cause chronic inflammation and toxic effects and can potentially interfere with the healing process [18], [19]. Besides these commercially available sealants, hydrogel adhesives are being developed and evaluated and show promising results for the application of cartilage repair [20], [21].

We definitely believe that a modified hedgehog-based triple fixation technique for treatment of a coronal shear injury of the trochlea is a suitable alternative compared to the existing fixation techniques with compression screws, Kirschner wires of headless Hebert screws. Especially in thin osteochondral injuries where stable fixation is challenging. Furthermore, the technique is quite easy to master when following the technical steps meticulously (Table 1).

**Table 1:** Advantages and pitfalls of a modified hedgehog-based fixation.

Modified hedgehog-based fixation	
Advantages	Pitfalls
Little collateral damage to the cartilage	Misfit of interlocking angles
Can be used in challenging thin osteochondral lesions	
Easy to master	
Stable fixation	

In conclusion, treating a patient with a dislocated coronal shear injury of the trochlea is challenging and needs both direct visualization of the fragment as well as stable fixation. In the present case, the anterior neurovascular approach seems elegant and provides adequate exposure. Furthermore, a modified hedgehog-based technique delivers a stable triple fixation of the osteochondral fragment.

## Provenance and peer review

Not commissioned, externally peer-reviewed.

## Consent

Written informed consent was obtained from the patient and his family for publication of this case report and accompanying images. A copy of the written consent is available for review by the Editor-in-Chief of this journal on request.

## Ethical approval

Patient and family approved this case report.

## Sources of funding

No sources of funding.

## Author contribution

M. Koëter: design, data analysis or interpretation, writing the paper.

P.P.W. van Hugten: design, data analysis or interpretation, writing the paper.

P.J. Emans: design, data interpretation, supervision of the paper.

J.A. Ten Bosch: design, data interpretation, supervision of the paper.

## Guarantor

M. Koëter.

## Research registration

1. Name of the registry: Researchregistry.com.
2. Unique identifying number or registration ID: Researchregistry6962 [22].
3. Hyperlink to your specific registration (must be publicly accessible and will be checked): https://www.researchregistry.com/browse-the-registry#home/registrationdetails/60e98968284519001f20064b/.

## Declaration of competing interest

All authors have nothing to declare.
